# Neural vulnerability to stress in adolescents: a longitudinal study using polyconnectomic scoring of depression risk

**DOI:** 10.1186/s12916-026-04704-3

**Published:** 2026-02-24

**Authors:** Yuan Liu, Meijuan Li, Chengfeng Chen, Shiying Wang, Ying Gao, Yifan Jing, Yan Zhou, Mengxin Xie, Changlin Zhang, Zhongchun Liu, Bin Zhang, Jie Li

**Affiliations:** 1 Department of Psychiatry, Renmin Hospital of Wuhan University, No. 99 Jiefang Road, Wuchang District, Wuhan, 430060 Hubei China; 2 Institute of Mental Health, Tianjin Anding Hospital, Mental Health Center of Tianjin Medical University, Liulin Rd, Hexi District, Tianjin, 300222 China; 3 Department of Psychiatry, The Affiliated Brain Hospital of Guangzhou Medical University, Guangzhou, China; 4 Taikang Center for Life and Medical Sciences, Wuhan University, Wuhan, China; 5 State Key Laboratory of Metabolism and Regulation in Complex Organisms, Wuhan University, Wuhan, China

**Keywords:** Adolescents, Perceived stress, Emotional distress, Cognitive performance, Polyconnectomic scoring

## Abstract

**Background:**

Adolescence is a sensitive period of brain development when perceived stress can shape emotional and cognitive outcomes. However, it remains unclear how neural vulnerability modulates these effects.

**Methods:**

In a longitudinal cohort of 407 adolescents, we assessed perceived stress, emotional symptoms, and cognitive function at baseline and follow-up. Neural vulnerability was indexed using polyconnectomic scoring for major depressive disorder (PCS-MDD), derived from large-scale functional connectivity patterns. An independent cohort of 80 adolescents with clinically diagnosed depressive disorders was included to explore the generalizability of the findings.

**Results:**

At baseline, perceived stress was strongly associated with anxiety (*β* = 0.59, *p* < 0.001) and depressive symptoms (*β* = 0.55, *p* < 0.001), and was modestly associated with lower performance across multiple cognitive domains. Longitudinally, increases in perceived stress were robustly associated with increases in anxiety (*β* = 0.56, *p* < 0.001) and depressive symptoms (*β* = 0.52, *p* < 0.001). Moreover, PCS-MDD significantly moderated these associations (anxiety symptoms: *p* = 0.025, *ΔR*^*2*^ = 0.008; depressive symptoms: *p* < 0.001, *ΔR*^2^ = 0.023). Additionally, baseline PCS-MDD was significantly associated with follow-up anxiety (*β* = 0.13, *p* = 0.024) and depressive symptoms (*β* = 0.11, *p* = 0.043). Exploratory analyses further indicated that perceived stress was most strongly associated with depressive symptoms among adolescents with clinically diagnosed depressive disorders and higher PCS-MDD.

**Conclusions:**

Higher PCS-MDD is associated with greater variability in stress-related emotional outcomes during adolescence. These results suggest that neural vulnerability may moderate the emotional correlates of stress and underscore the potential relevance of personalized interventions targeting stress regulation in youth with elevated vulnerability profiles.

**Supplementary Information:**

The online version contains supplementary material available at 10.1186/s12916-026-04704-3.

## Background

Adolescence is a sensitive period of emotional and cognitive development, during which perceived stress can have profound effects [1, 2]. Perceived stress refers to the degree to which individuals appraise life situations as unpredictable, uncontrollable, or overwhelming, irrespective of objective stressors [3]. It has long been recognized as a key risk factor for the onset and exacerbation of emotional disorders, particularly anxiety and depression [4, 5]. Among these, major depressive disorder (MDD) is one of the most notable stress-related psychiatric conditions in adolescence, with increasing evidence suggesting that chronic stress plays a central role in its development and progression [6, 7]. In addition, stress-related emotional symptoms frequently co-occur with potential vulnerabilities in cognitive domains such as attention, executive function, and processing speed, further underscoring heterogeneity in neurodevelopmental risk [8]. However, the underlying mechanisms linking stress to emotional and cognitive dysfunction remain poorly understood, especially in adolescent populations.

Neuroimaging research has highlighted individual variability in susceptibility to stress-related psychopathology [9, 10], with functional brain activity emerging as a promising biomarker [11, 12]. Existing approaches, such as Connectome-based Predictive Modeling (CPM) [13, 14] and Functional Segregation Analysis (FSA) [15, 16], have considerably enhanced our comprehension of the relationship between functional connectivity and both behavioral and clinical manifestations. Nonetheless, these techniques are primarily designed to predict behavioral variation or to characterize specific network properties, and therefore target analytical aims that differ from those involved in assessing an individual’s similarity to disease-related connectomic patterns.

One innovative approach that addresses this gap is polyconnectomic scoring (PCS), which quantifies the alignment of an individual’s brain functional connectivity patterns with disease-specific connectivity templates [17]. This template-based framework offers a complementary perspective on neural vulnerability, providing a single, interpretable metric that reflects system-level resemblance to MDD-related connectivity architecture. Specifically, PCS for MDD (PCS-MDD) indexes the degree to which an individual’s brain functional connectivity pattern resembles the connectivity template previously associated with MDD. Previous evidence indicated that higher PCS-MDD values have been linked to poorer cognitive performance, alongside heightened neuroticism, increased anxiety, and greater reliance on mental health services [17]. Together, these findings suggest that PCS-MDD may index stress-related neural vulnerability across emotional and cognitive domains.

While promising, the utility of PCS-MDD has not yet been validated in adolescent populations, where neurodevelopmental processes and stress susceptibility may differ from adults [18, 19]. This gap limits our understanding of whether PCS-MDD captures early markers of risk relevant to youth mental health, particularly as adolescent vulnerability may emerge through subtle, distributed connectivity deviations [20, 21] that a template-based metric is well positioned to detect.

**Fig. 1:**
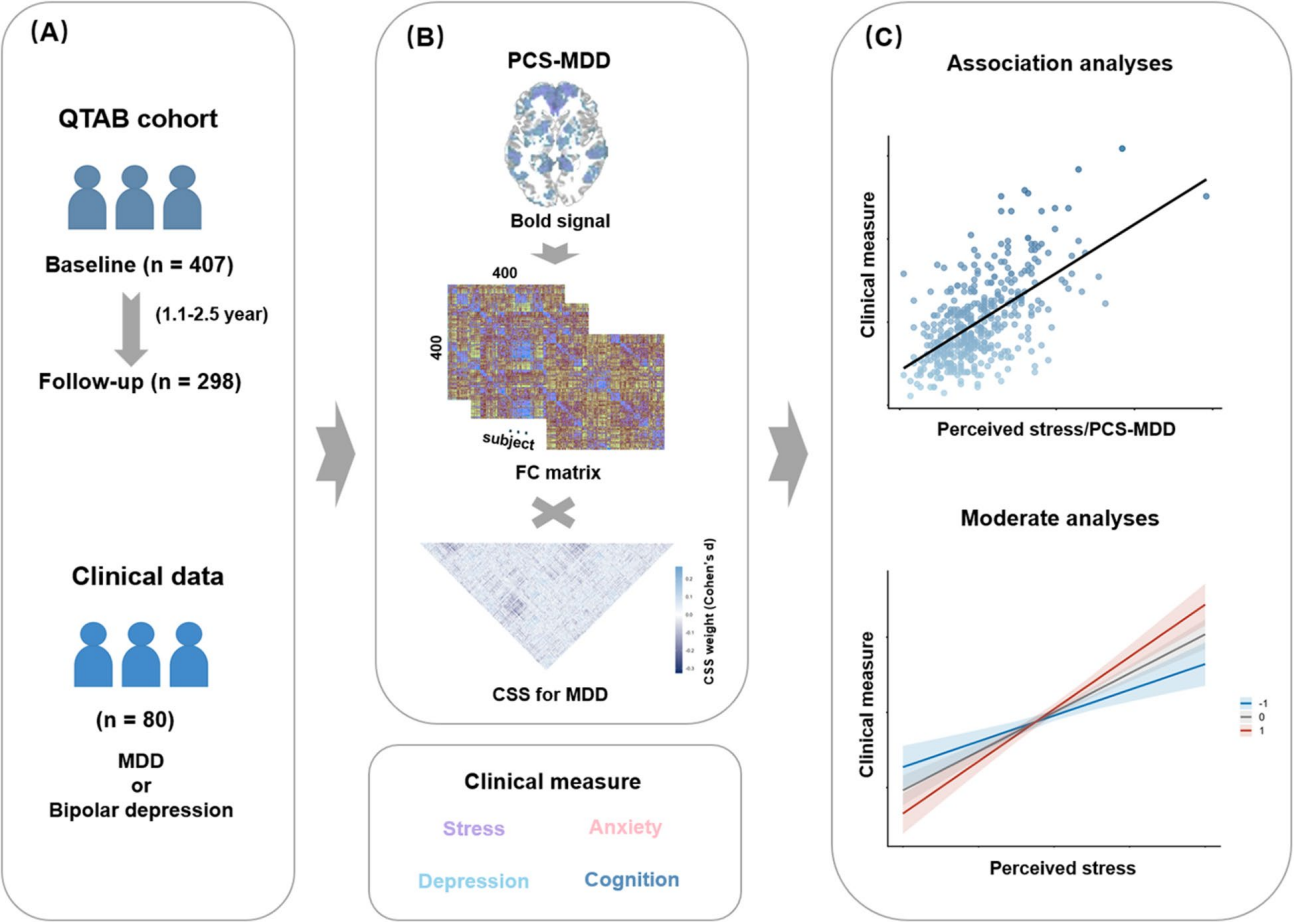
Overview of the study design. **A** Participants’ information: The QTAB adolescent cohort included 407 participants at baseline and 298 at follow-up. An independent clinical sample included 80 adolescent patients (MDD or bipolar depression). **B** PCS-MDD and symptom/cognitive measures: Each participant underwent resting-state fMRI, along with standardized assessments of perceived stress, emotional symptoms (depression and anxiety), and cognitive performance. Individual BOLD signals were transformed into 400 × 400 functional connectivity matrices and weighted against the CSS for MDD template to compute a PCS-MDD score for each participant. In the template, color denotes the CSS weight (Cohen’s d) for each connection, with deep blue indicating negative values and light blue indicating positive values. **C** Association Analyses and Moderate Analyses. Abbreviations: QTAB, Queensland Twin Adolescent Brain project; MDD, major depressive disorder; PCS-MDD, polyconnectomic scoring for major depressive disorder; BOLD, blood oxygen level-dependent; FC, functional connectivity; CSS, connectome summary statistics

To address these gaps, this study had three primary aims. First, we examined whether PCS-MDD moderates the associations between perceived stress, emotional symptoms (anxiety and depression), and cognitive performance in a large adolescent cohort. Second, we investigated whether baseline PCS-MDD shapes the longitudinal associations between changes in perceived stress and changes in emotional symptoms and cognitive performance. Third, we explored the clinical generalizability in an independent sample of adolescents with clinically diagnosed depressive disorders (Fig. 1). We hypothesized that higher perceived stress would be associated with greater emotional symptoms and poorer cognitive performance, and that PCS-MDD would moderate the detrimental effects of perceived stress on these outcomes.

## Methods

### Experimental model and participants details

The study included 407 participants (aged 9 to 13 years) from the Queensland Twin Adolescent Brain (QTAB) project [22], a community-based cohort of typically developing children. Of the 422 participants initially enrolled, five were excluded due to magnetic resonance imaging (MRI) intolerance and ten were excluded due to missing functional magnetic resonance imaging (fMRI) data. No additional exclusions were made beyond missing fMRI data, and participants with complete fMRI, symptom, and cognitive data were retained for the final analyses (detail in Additional file 1: Methods). Written informed consent was obtained from all participating families prior to inclusion in the study. The study was conducted in accordance with approvals obtained from the Children’s Health Queensland Human Research Ethics Committee (HREC) and the University of Queensland HREC.

Baseline measures included structural and functional brain imaging, as well as assessments of cognition, anxiety and depressive symptoms, and stress levels. After an average follow-up period of 20 months, participants (*n* = 298) returned for a second assessment, which included repeated evaluations of cognition, anxiety and depressive symptoms, and stress levels.

### Imaging phenotypes and preprocessing

Structural and functional scans were acquired using a 3 T Magnetom Prisma scanner (Siemens Medical Solutions, Erlangen). All MRI data were collected using identical scanner hardware and acquisition platforms at a unique site. Detailed scanning parameters for resting-state fMRI and 3D T1-weighted images are provided in Additional file 1: Methods.

Image preprocessing was conducted using Data Processing Assistant for Resting-State fMRI (DPARSF) toolbox (http://www.rfmri.org/) [23]. Preprocessing steps included discarding the first 10 volumes, head motion correction, covariate regression, spatial normalization to the Montreal Neurological Institute (MNI) template, and bandpass filtering (0.01–0.1 Hz), as described in detail in Additional file 1: Methods.

### PCS-MDD

#### PCS-MDD calculation

Functional connectivity maps were constructed by analyzing the average blood oxygen level-dependent (BOLD) signal across brain regions defined by the Schaefer 400-parcel atlas [24]. First, mean BOLD signals were extracted from the preprocessed fMRI data for each of the 400 regions. Then, Pearson correlation coefficients were calculated between the mean BOLD signals of all region pairs, resulting in 400 × 400 functional connectivity maps for each participant. Consistent with the PCS calculations pipeline for the previous study [17], these correlations were used directly without Fisher’s Z transformation, and no covariates were regressed from the connectivity matrices prior to scoring to preserve the standardized data structure across participants.

PCS-MDD was calculated using the PCS toolbox [17], which includes meta-analytic connectome summary statistics (CSS) maps for various conditions, including MDD (https://github.com/dutchconnectomelab/pcs-toolbox.git). For this study, the CSS map for MDD (fc_depression_schaefer400-yeo7_gmean) was selected based on prior recommendations [17], derived from four cohorts including 884 MDD patients and 1,605 controls (Additional file 1: Table S1; Figure S1). The PCS-MDD for each participant was computed as the weighted average of the MDD-specific CSS values and the individual’s functional connectivity map (Fig. 1). Specifically, PCS-MDD was calculated as:

$$PCS-MDD= \frac{1}{n}\sum_{i }^{n}({\beta }_{i}\times {C}_{i})$$

where ${C}_{i}$ is the individual’s functional connectivity value for connection $i$, and ${\beta }_{i}$ is the corresponding MDD-specific CSS weight. Here, $n$ denotes the number of connections with non-zero CSS weights, such that only connections contributing information in the MDD meta-analytic map are included in the averaging. No additional scaling was applied beyond the standardization intrinsic to the meta-analytic CSS weights. These scores quantify the degree of similarity between an individual’s brain functional connectivity pattern and an independently derived MDD-related connectomic template. They reflect a continuous measure of pattern alignment, without implying diagnostic status or discrete biological categories. Higher PCS-MDD values indicate stronger alignment with the MDD-related connectomic pattern, whereas lower values indicate weaker alignment. Accordingly, PCS-MDD values should be interpreted as relative indices of pattern alignment rather than absolute measures of depression risk or severity.

#### CSS for PCS-MDD

To enhance interpretability, we further quantified contribution patterns in the CSS template. At the edge level, we extracted CSS weights for all region-to-region connections to identify the strongest positive and negative contributors (e.g., top 10/20 edges). At the network level, CSS weights were aggregated according to the Yeo 7-network parcellation to characterize within- and between-network contributions. All contribution analyses were performed exclusively on the CSS template rather than on the present sample. Detailed results of these analyses, including edge- and network-level contribution maps, are reported in Additional file 1: Results; Table S2; Figure S2.

### Emotional symptom measures

#### Perceived stress

Structured assessments were conducted to evaluate perceived stress using the Daily Life Stressors Scale (DLSS). The DLSS is a 30-item questionnaire designed to measure the severity of aversive feelings and daily life challenges in young individuals [25]. Higher scores on the DLSS indicate greater levels of perceived stress.

#### Anxiety and depression

Depressive symptoms were assessed using the self-reported Short Moods and Feelings Questionnaire (SMFQ) designed to evaluate depressive symptoms in children and adolescents [26]. Anxiety symptoms were measured using the Spence Children’s Anxiety Scale (SCAS), a widely utilized tool for assessing anxiety disorder symptoms in children and adolescents [27].

### Cognitive measures

Cognitive performance was evaluated using the iPad version of the National Institutes of Health Toolbox Cognition Battery (NIHTB-CB), covering executive function, memory, language, and pattern comparison [28]. All scores were age-adjusted and standardized. This battery is widely adopted in adolescent cohorts [29, 30]. A full description of the Toolbox tasks, including administration procedures and domain-specific scoring, is provided in Additional file 1: Methods.

### Main analyses

All statistical analyses were conducted using SPSS 26.0 (Chicago, USA) and R 4.5.2 in RStudio. Data were presented as mean ± standard deviation (SD) or ratios, with statistical significance set at a two-tailed *p*-value < 0.05. Bonferroni correction was applied to all association analyses to account for multiple comparisons.

#### Baseline analyses

1. Associations between perceived stress and emotional symptoms/cognitive performance
  Linear mixed‐effects models (LMMs) were conducted to evaluate the associations between perceived stress (DLSS score) and outcome measures, including anxiety symptoms (SCAS score), depressive symptoms (SMFQ score), and cognitive performance (six NIHTB-CB subscale scores). Age and sex were included as fixed-effect covariates, and family structure was modeled with a random intercept to account for within-family dependence.
2. Associations between PCS-MDD and emotional symptoms/cognitive performance
  LMMs assessed the associations between PCS-MDD and outcome measures, including anxiety symptoms (SCAS score), depressive symptoms (SMFQ score), and cognitive performance (six NIHTB-CB subscale scores). Age, sex, and mean framewise displacement (FD) were included as fixed-effect covariates, and family structure was modeled with a random intercept to account for within-family dependence.
3. PCS-MDD moderates the associations between perceived stress and emotional symptoms/cognitive performance
  Moderation analyses were performed using LMMs, where PCS-MDD (W) was modeled as a continuous moderator of the association between DLSS (X) and outcome measures (Y). Age, sex, and mean FD were included as fixed-effect covariates, and family structure was modeled using a random intercept to account for within-family clustering. All models included mean-centered continuous variables and tested the conditional effects of DLSS at ± 1 SD of PCS-MDD. Significance of interaction terms was evaluated using 95% confidence intervals and *ΔR*^2^ change.

#### Follow-up analyses

1. Change score computation
  Change scores were calculated for perceived stress (DLSS score), anxiety (SCAS score), depression (SMFQ score), and cognitive performance (six NIHTB-CB subscale scores) by subtracting baseline values from follow-up values.
2. Associations between change in perceived stress and emotional symptoms/cognitive performance
  LMMs examined the associations between changes in perceived stress (DLSS change scores) and changes in anxiety (SCAS change scores), depression (SMFQ change scores), and cognitive performance (NIHTB-CB subscale change scores). Sex and follow-up duration (calculated as the difference between follow-up age and baseline age) were included as fixed-effect covariates, and family structure was modeled with a random intercept to account for within-family dependence.
3. Baseline PCS-MDD moderates the associations between change in perceived stress and emotional symptoms/cognitive performance
  Moderation analyses were performed using LMMs, where PCS-MDD (W) was modeled as a continuous moderator of the association between DLSS change (X) and outcome measures change (Y). Follow-up duration, sex, and mean FD were included as fixed-effect covariates, and family structure was modeled using a random intercept to account for within-family clustering. All models included mean-centered continuous variables and tested the conditional effects of DLSS change at ± 1 SD of PCS-MDD. Significance of interaction terms was evaluated using 95% confidence intervals and *ΔR*^2^ change.
4. Longitudinal associations between baseline PCS-MDD and follow-up emotional symptoms and cognitive performance
  LMMs assessed whether baseline PCS-MDD was associated with outcome measures, including anxiety symptoms (SCAS score), depressive symptoms (SMFQ), and cognitive performance (NIHTB-CB subscale change scores) at follow-up. Age, sex, and mean FD were included as fixed-effect covariates, and family structure was modeled with a random intercept to account for within-family dependence.

### Sensitivity analyses

To determine whether the associations between PCS-MDD and anxiety/depressive symptoms reflected template-specific effects rather than nonspecific dimensional or methodological influences, we re-estimated all primary moderation and association models using alternative disorder-specific templates, including the polyconnectomic scoring for anxiety disorder (PCS-Anxiety), bipolar disorder (PCS-Bipolar), and schizophrenia (PCS-SCZ).

### Exploratory analyses

This study explored the association between perceived stress and depressive symptoms in adolescents with clinically diagnosed depressive disorders (MDD and bipolar depression) and examined whether neural vulnerability moderates these associations. The independent clinical cohort comprised 80 adolescents aged 12–18 years, recruited from Tianjin Anding Hospital, all of whom were diagnosed using the Mini International Neuropsychiatric Interview for Children and Adolescents (M.I.N.I. Kid) 5.0 Revision and had a Montgomery–Åsberg Depression Rating Scale (MADRS) score ≥ 22. Exclusion criteria encompassed psychotic symptoms, severe neurological or medical conditions, recent brain stimulation, and inability to cooperate (see Additional file 1: Methods).

All participants underwent fMRI scans and completed assessments for perceived stress and emotional distress (detailed in Additional file 1: Methods). To minimize site-related variability and enhance comparability with the QTAB cohort, all clinical imaging data were collected at a single site using the same scanner model and acquisition platform as QTAB (detailed parameters provided in Additional file 1: Table S3). Consistent with the QTAB pipeline, all data were preprocessed using the same standardized procedures to ensure reproducibility and harmonization across cohorts. PCS-MDD scores were computed using the same algorithm and parameters as in the main analyses, ensuring full cross-cohort comparability. Perceived stress was evaluated using the Perceived Stress Scale-14 (PSS-14), while depressive symptoms were assessed with MADRS. The study protocol was approved by the Ethics Committee of Tianjin Anding Hospital, and written informed consent was obtained from all parents or guardians.

We first explored the association between perceived stress (PSS-14)/PCS-MDD and depressive symptoms (MADRS) using linear regression models, with sex, age, and mean FD (only for PCS-MDD) included as covariates. All continuous variables were mean-centered and standardized. We then explored whether PCS-MDD moderated this association by including the PSS-14 × PCS-MDD interaction term in the model and evaluating its significance along with the change in explained variance (ΔR^2^). To explore the association between perceived stress and depressive symptoms across different neural vulnerability levels, participants were divided into three balanced groups (low, medium, high) [31] based on PCS-MDD scores for subgroup analysis.

To further explore whether PCS-MDD scores differed between the QTAB and clinical validation samples after accounting for demographic variables, we performed an analysis of covariance (ANCOVA), with PCS-MDD as the dependent variable, group as the fixed factor, and age and sex as covariates.

## Results

### Demographic, symptom, and cognitive characteristics

The study cohort consisted of 407 participants with a female-to-male ratio of 197/210 and a mean age of 11.32 years. Handedness distribution included 72 left-handed and 335 right-handed participants. Emotional symptoms included a mean DLSS score of 22.10, a mean SCAS score of 24.79, and a mean SMFQ score of 4.25. For cognitive performance, the mean scores on the six subscales of NIHTB-CB were Flanker (98.27), Card Sort (100.87), Pattern Comparison (98.55), Picture Sequence (101.37), Picture Vocabulary (103.72), and Oral Reading Recognition (106.18) (Table 1).

**Table 1:** Demographic, symptom, and cognitive characteristics in QTAB cohort. Abbreviations: *QTAB*, Queensland Twin Adolescent Brain project; *DLSS*, Daily Life Stressors Scale; *SCAS*, Spence Children’s Anxiety Scale; *SMFQ*, Short Moods and Feelings Questionnaire

Characteristics	QTAB cohort (*n* = 407)
Sex (female/male)	197/210
Age (years)	11.32 ± 1.34
Handedness (left/right)	72/335
Stress	
DLSS score	22.10 ± 11.63
Anxiety and depression	
SCAS score	24.79 ± 13.94
SMFQ score	4.25 ± 3.47
Cognition	
Flanker	98.27 ± 15.47
Card sort	100.87 ± 19.84
Pattern comparison	98.55 ± 21.25
Picture sequence	101.37 ± 16.16
Picture vocabulary	103.72 ± 13.56
Oral reading recognition	106.18 ± 15.97

### Baseline analyses

#### Associations between perceived stress and emotional symptoms/cognitive performance

**Fig. 2:**
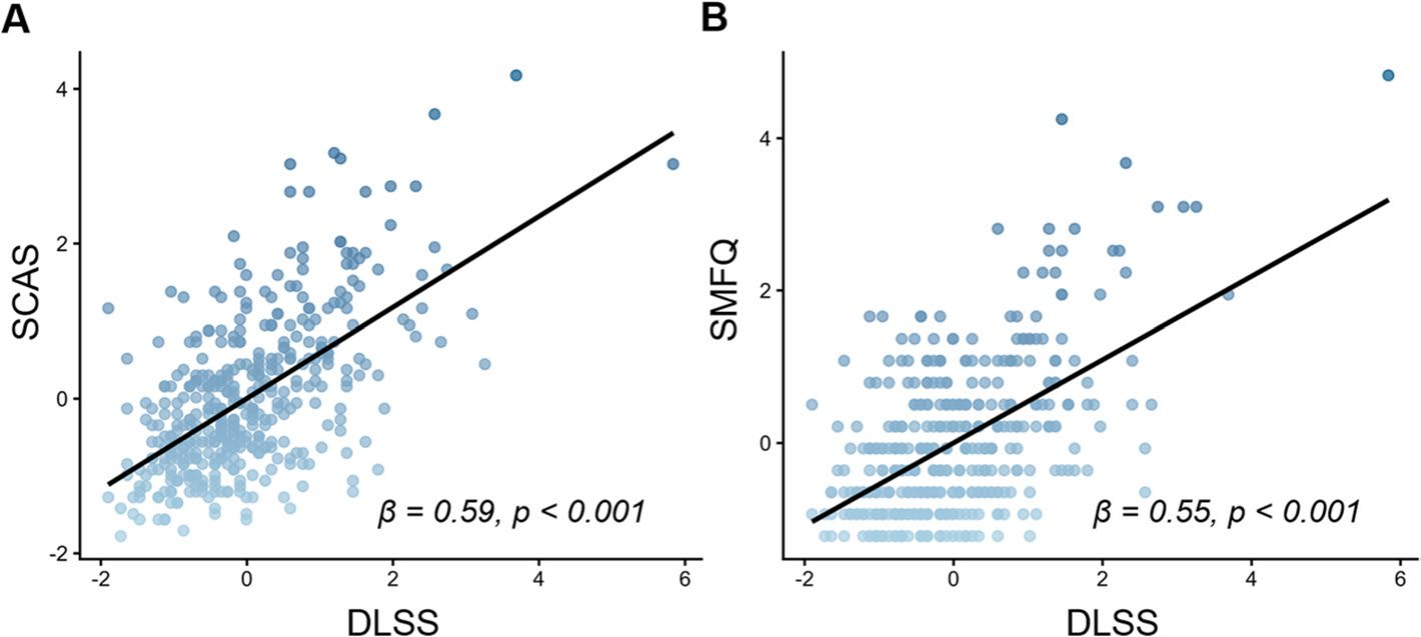
Associations between perceived stress and anxiety/depressive symptoms at baseline. Abbreviations: SCAS, Spence Children’s Anxiety Scale; SMFQ, Short Moods and Feelings Questionnaire; DLSS, Daily Life Stressors Scale

Significant positive associations were observed between DLSS scores and both SCAS (*β* = 0.59, *p* < 0.001) and SMFQ (*β* = 0.55, *p* < 0.001) (Fig. 2). After applying Bonferroni correction, significant associations persisted between DLSS and SCAS and SMFQ. Perceived stress showed modest associations with cognitive measures, and none survived Bonferroni correction (Additional file 1: Results; Table S4).

#### Associations between PCS-MDD and emotional symptoms/cognitive performance

No significant associations were observed between PCS-MDD and SCAS or SMFQ scores (*p* > 0.05) (Table S6). Similarly, PCS-MDD showed no robust associations with cognitive measures after Bonferroni correction (Additional file 1: Results; Table S5).

#### PCS-MDD moderates the associations between perceived stress and emotional symptoms/cognitive performance

No significant moderation effects were found for SCAS, SMFQ, and NIHTB-CB subscale (all *p* > 0.05).

### Follow-up analyses

Participant age at baseline and follow-up, as well as the interval between assessments, is shown in Additional file 1: Figure S3. Demographic, symptom, and cognitive characteristics at both time points are summarized in Additional file 1: Table S6.

#### Change scores in perceived stress, emotional symptoms, and cognitive performance

At follow-up, perceived stress (DLSS scores) and depressive symptoms (SMFQ scores) showed significant increases compared to baseline, while anxiety symptoms (SCAS scores) remained unchanged (Additional file 1: Figure S4). Full cognitive change score results are reported in Additional file 1: Results; Figure S4.

#### Associations between changes in perceived stress and emotional symptoms/cognitive performance

**Fig. 3:**
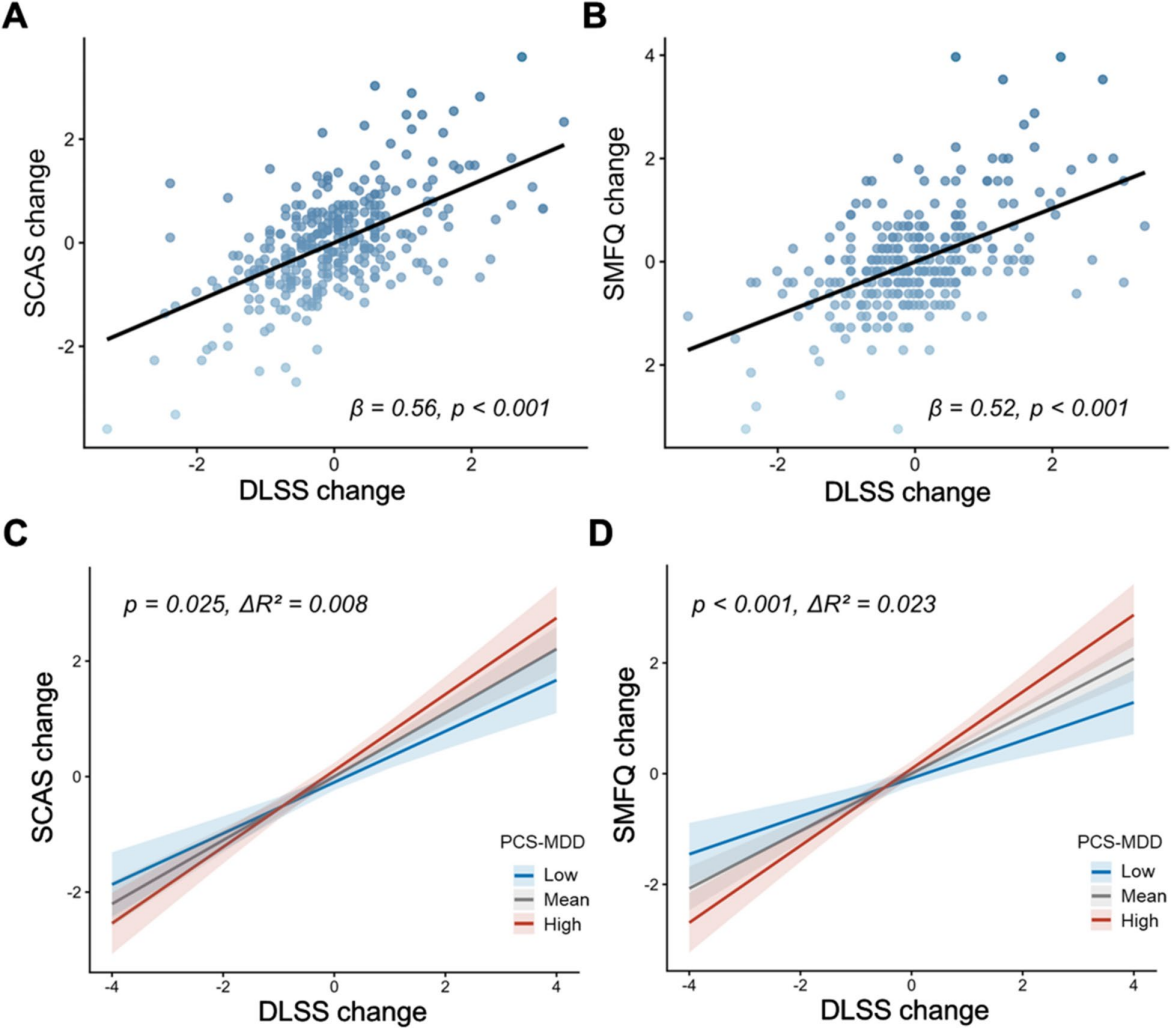
Associations between changes in perceived stress and anxiety/depressive symptoms, and the moderating role of PCS-MDD. **A**–B Associations between changes in perceived stress and anxiety/depressive symptoms. **C**–**D** PCS-MDD moderates the associations between changes in perceived stress and anxiety/depressive symptoms. Abbreviations: SCAS, Spence Children’s Anxiety Scale; SMFQ, Short Moods and Feelings Questionnaire; DLSS, Daily Life Stressors Scale; PCS-MDD, polyconnectomic scoring for major depressive disorder

LMMs indicated strong associations between changes in perceived stress and emotional symptoms but not cognitive outcomes. Increases in DLSS scores were positively correlated with changes in SCAS (*β* = 0.56, *p* < 0.001) and SMFQ scores (*β* = 0.52, *p* < 0.001) (Fig. 3A–B). These associations remained significant after Bonferroni correction. No significant associations were found between changes in perceived stress and changes in cognitive performance (all *p* > 0.05) (Additional file 1: Table S7).

#### Baseline PCS-MDD moderates the associations between change in perceived stress and emotional symptoms/cognitive performance

The moderation analysis revealed that PCS-MDD significantly moderated the relationship between DLSS change and SCAS change (*β* = 0.11, 95% CI (0.01, 0.21), *p* = 0.025; *ΔR*^2^ = 0.008) and SMFQ change (*β* = 0.18, 95% CI (0.08, 0.27), *p* < 0.001; *ΔR*^2^ = 0.023) (Fig. 3C–D). After Bonferroni correction (0.05/2), the moderation effect for DLSS–SMFQ changes remained significant. No significant moderation effects were found for cognitive performance (all *p* > 0.05) (Additional file 1: Table S8).

#### Associations between baseline PCS-MDD and follow-up emotional symptoms and cognitive performance

**Fig. 4:**
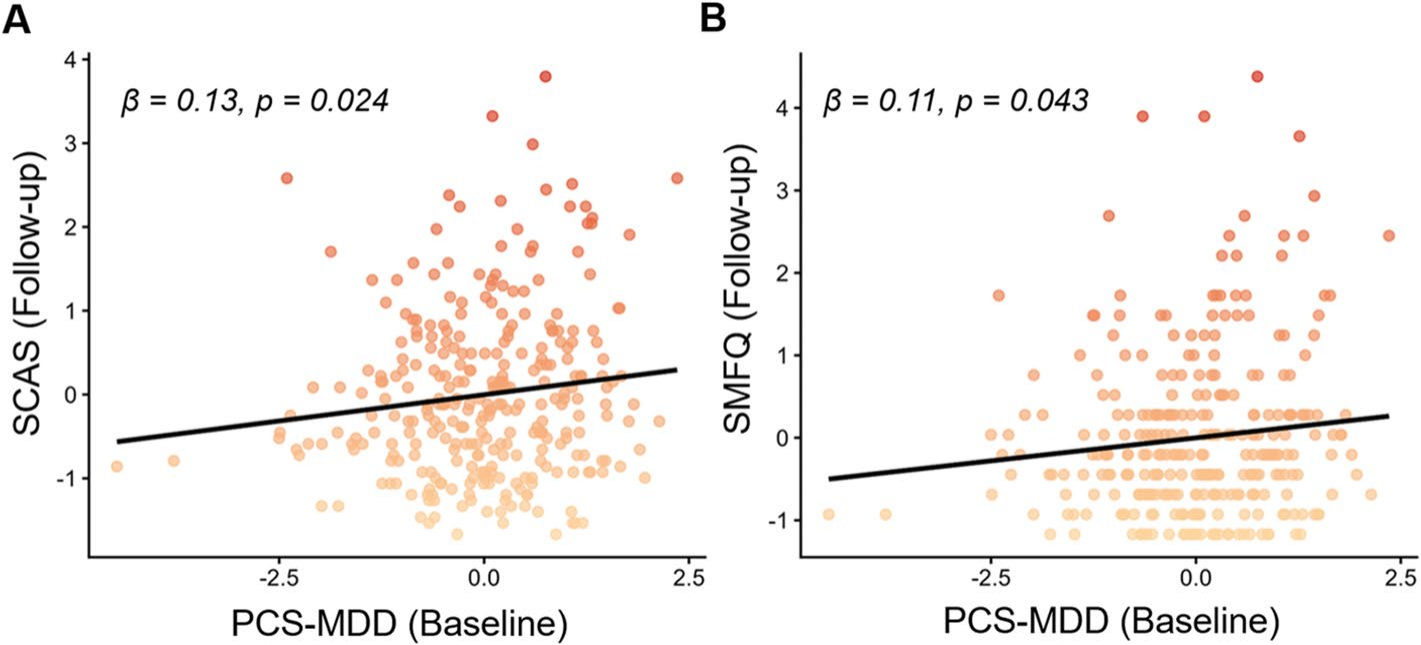
Associations between baseline PCS-MDD and anxiety/depressive symptoms at follow-up. Abbreviations: SCAS, Spence Children’s Anxiety Scale; SMFQ, Short Moods and Feelings Questionnaire; PCS-MDD, polyconnectomic scoring for major depressive disorder

Significant positive associations were observed between baseline PCS-MDD and both SCAS (*β* = 0.13, *p* = 0.024) and SMFQ (*β* = 0.11, *p* = 0.043) at follow-up (Fig. 4). After applying the Bonferroni correction (0.05/2) and additional controlling for baseline anxiety and depressive symptoms, the association between baseline PCS-MDD and SCAS at follow-up remained significant. No significant associations were observed for cognitive outcomes after correction (Additional file 1: Results; Table S9).

### Sensitivity analyses

In the sensitivity analyses employing alternative disorder-specific templates (PCS-Anxiety, PCS-Bipolar, and PCS-SCZ) (Additional file 1: Table S10–S13), none of these templates significantly moderated the relationship between changes in perceived stress and changes in depressive symptoms (all *p* > 0.05) (Additional file 1: Table S12). Similarly, none of the alternative templates was significantly associated with depressive symptoms at follow-up (all *p* > 0.05) (Additional file 1: Table S13).

### Exploratory analyses

The clinical data included 80 participants (61 females, 19 males; 63 MDD, 17 bipolar depression) with a mean age of 14.91 years. Key clinical measures included perceived stress (PSS-14: 54.48) and depressive symptoms (MADRS: 29.03) (Additional file 1: Table S14–S15).

In clinical sample, PSS-14 scores showed a significant positive association with MADRS scores (*β* = 0.36, *p* < 0.001). Conversely, PCS-MDD did not demonstrate a significant association with MADRS scores, nor did it serve as a moderator of the relationship between PSS-14 and MADRS scores (both *p* > 0.05). Subgroup analyses revealed a positive association between PSS-14 and MADRS scores across PCS-MDD levels, with the strongest association observed in the high PCS-MDD subgroup (*β* = 0.57, *p* = 0.020), while correlations in the low (*β* = 0.17, *p* = 0.238) and medium subgroups (*β* = 0.31, *p* = 0.072) were not statistically significant (Additional file 1: Figure S5).

Participants in the clinical data were significantly older and had a higher proportion of females (all *p* < 0.001) compared to the QTAB cohort. Importantly, PCS-MDD scores were significantly higher in the clinical data than in the QTAB sample (*p* = 0.003), and this difference remained significant after controlling for age and sex using ANCOVA (Additional file 1: Figure S5).

## Discussion

This study is the first, to our knowledge, to investigate the associations between perceived stress, PCS-MDD, and emotional and cognitive outcomes in adolescents. Our findings suggest a prominent association between perceived stress and emotional symptoms, and further indicate that inter-individual differences in PCS-MDD are associated with variability in stress-related emotional responses. Moreover, PCS-MDD was associated with subsequent emotional symptoms over time. Taken together, these results suggest that PCS-MDD may index individual differences in neural vulnerability relevant to stress-related mood outcomes, rather than serving as a deterministic risk marker.

Our findings reveal a robust positive association between perceived stress and both anxiety and depressive symptoms. This is consistent with prior research that identifies stress as a key risk factor for internalizing symptoms in youth [32]. Adolescence is a critical period of heightened stress sensitivity and developing emotional and regulatory systems, making stress especially impactful [33]. High stress levels can disrupt emotion regulation, increase negative emotions, and heighten the risk of anxiety and depression [34]. In contrast, the relationship between perceived stress and cognitive performance was weak and mostly diminished after adjustments. While stress affects cognitive development, particularly in processing speed and memory [35, 36], these effects were less pronounced than those on emotional symptoms. Overall, these findings highlight the relative salience of stress for emotional, rather than cognitive, outcomes during adolescence. However, in this cross-sectional analysis, associations between PCS-MDD and emotional or cognitive outcomes were weak, and PCS-MDD did not significantly moderate the link between perceived stress and emotional symptoms, which may partly reflect the dynamic and heterogeneous nature of early adolescent neurodevelopment [37].

Longitudinal analyses revealed a notable increase in perceived stress and depressive symptoms over time, while anxiety levels remained stable. Cognitive performance exhibited mixed trajectories, with improvements in specific domains, such as executive functioning and processing speed, alongside declines in vocabulary-related tasks. This domain-specific pattern of cognitive change likely reflects the heterochronic maturation of different cognitive systems during adolescence [2, 38].

Longitudinal association analyses demonstrated that increases in perceived stress were positively associated with increases in anxiety and depressive symptoms over time, supporting a developmental trajectory perspective in which stress remains associated with adolescent mental health over time [39]. In addition, moderation analyses revealed a significant interaction between PCS-MDD and changes in perceived stress in relation to changes in depressive symptoms. Specifically, adolescents with higher PCS-MDD showed stronger associations with increases in anxiety and depressive symptoms along with rising perceived stress over time. Although the moderation effect size was small, it remained significant after correcting for multiple comparisons, suggesting that even modest differences in neural vulnerability may be associated with stress-related mood dysregulation during development [40]. Together, these findings provide converging evidence that individual differences in large-scale functional brain architecture are associated with variability in stress-related emotional difficulties. Notably, considering that early and mid-adolescence are characterized by rapid neurocognitive and socioemotional development [41], the observed patterns may partly reflect normative developmental shifts that interact with individual differences in neural vulnerability. In addition, variability in participants’ age and in the length of follow-up intervals may have influenced the magnitude or detectability of stress-related changes and neural vulnerability effects [42, 43]. Although the precise impact cannot be quantified, these factors likely contribute to individual differences in observed longitudinal outcomes and underscore the need for developmentally stratified or more densely sampled designs in future studies. In this context, the absence of robust longitudinal associations with cognition should be interpreted cautiously, as developmental compensation, practice effects from repeated testing, and the limited sensitivity of broad screening tools like NIHTB-CB may mask subtle stress-related cognitive changes [44–46]. Notably, adolescence involves substantial neurobiological changes, including hormonal shifts and maturation of emotion-regulation circuits, which may interact with neural vulnerabilities to influence depression risk [47, 48]. These interactions likely involve multiple overlapping mechanisms, rather than a single pathway, reflecting the concept of degeneracy in brain-behavior relationships.

Furthermore, baseline PCS-MDD was significantly associated with follow-up anxiety and depressive symptoms, consistent with the interpretation of PCS-MDD as indexing individual differences in neural vulnerability that may vary across developmental periods. Sensitivity analyses using alternative disorder-specific templates revealed no meaningful associations with follow-up depressive symptoms and no evidence of moderation of the stress–depressive change relationship, providing tentative support for the relative specificity of PCS-MDD. However, these null findings should be interpreted with caution given their limited scope. Derived from an MDD-specific CSS template, PCS-MDD is influenced by negative intra-network connections within the somatomotor network and positive inter-network connections between the somatomotor and salience/attention networks. These networks are central to established neurobiological models of MDD, including stress–diathesis and salience-network dysfunction frameworks [49, 50]. Within these models, somatomotor network alterations are viewed as downstream psychomotor and embodied expressions of stress-related affective dysregulation, whereas aberrant salience/attention network engagement contributes to heightened sensitivity to stress and threat-related cues. The somatomotor–salience/attention interactions indicated by PCS-MDD align with core elements of these frameworks: abnormal salience processing, increased threat response, and impaired emotion regulation, all consistently linked to depressive symptoms [51, 52]. Importantly, these network patterns should be interpreted as conceptually corresponding to established models of mood dysregulation, rather than as evidence of a direct mechanistic pathway. The observed longitudinal associations are correlational and may potentially reflect interactions between these vulnerability-related connectivity patterns and ongoing neurodevelopmental processes. Taken together, PCS-MDD should be interpreted not as a fixed, trait-like marker, but as a dynamic, state-dependent neural signature indexing a developmentally contingent expression of depression-related neural vulnerability.

Exploratory analyses showed a significant positive correlation between perceived stress and depressive symptoms in adolescents with clinically diagnosed depressive disorders, supporting stress as a key risk factor for emotional distress during this developmental period. These findings highlight the potential relevance of stress management interventions to improve emotional well-being in both clinical and non-clinical adolescent populations. Cross-sectional moderation analyses, consistent with the QTAB cohort, did not reveal a clear PCS-MDD × stress interaction. In subgroup analyses, the strongest stress–depression association was observed in adolescents with high PCS-MDD scores, suggesting a potential link between heightened neural vulnerability and stress-related mood disturbances [47, 49]. However, these findings are exploratory and should be interpreted with caution. Notably, PCS-MDD scores were significantly higher in the clinical cohort compared to the community-based QTAB sample, even after controlling for age and sex, suggesting its potential group-level differentiation between adolescents with mood disorders and community participants. Although demographic and methodological differences between cohorts preclude direct comparisons or formal discriminant validity testing, these findings offer preliminary support for the external applicability of PCS-MDD as a neural vulnerability index in clinical populations.

Several limitations of this study should be acknowledged. First, self-reported stress, anxiety, and depression can lead to biased responses and overlapping concepts, possibly exaggerating their links, so findings should be interpreted cautiously. Second, while PCS-MDD provides a promising neurobiological framework, it is an exploratory, correlational index and does not support causal inference. It also does not incorporate other potential moderators (e.g., genetic factors, environmental influences, or comorbid conditions) that may contribute to individual differences in stress susceptibility. Third, although the study includes longitudinal data, the observational nature of the design precludes causal inferences about the directionality of associations between PCS-MDD, stress, and mental health outcomes. Moreover, the absence of intermediate stress assessments and the two-wave design limit our ability to examine temporal fluctuations in stress and to fully characterize developmental trajectories. Meanwhile, the relatively broad age range of participants (9–16 years) and variability in follow-up intervals may have introduced heterogeneity in developmental stage, potentially influencing the magnitude and detectability of stress- and neural vulnerability-related effects. Future studies incorporating experimental or intervention-based designs, or employing developmentally stratified and more densely sampled longitudinal designs, are needed to clarify causal mechanisms and better account for developmental influences. Fourth, as both cohorts were acquired at single sites, cross-site stability of PCS-MDD could not be assessed; multi-site validation with harmonization methods is needed to establish its robustness. Fifth, the cross-sectional clinical cohort could not reproduce the longitudinal moderation effect. Finally, sample differences in sex, age, country, cultural background, and symptom measures limit cross-cohort comparability.

## Conclusions

This study highlights the association between perceived stress and emotional and cognitive outcomes during adolescence, a sensitive period of development. Importantly, our findings indicate that individual differences in PCS-MDD are associated with variability in the strength of the longitudinal association between increases in perceived stress and changes in emotional symptoms. Adolescents with higher PCS-MDD scores tended to show stronger coupling between rising stress and worsening emotional symptoms. This pattern is consistent with the conceptualization of PCS-MDD as a potential index of neural vulnerability related to stress processing. Taken together, these findings highlight the potential relevance of stress-focused approaches for supporting adolescent mental health, while underscoring the need for future studies to establish causal mechanisms and intervention implications.

## Supplementary Information

Additional file 1: Supplementary methods, results, tables, and figures. Supplementary methods summarize participant characteristics, assessments, and MRI procedures. Supplementary results present analyses of CSS contribution patterns, PCS-MDD distributions, and cognitive associations. Table S1: Demographic characteristics of participants across four MDD cohorts; Table S2: Top 10 positive and negative connection weights in the MDD-specific CSS map; Table S3: MRI acquisition key parameters for the QTAB and clinical samples; Table S4: Associations between perceived stress and cognitive performance at baseline; Table S5: Associations between PCS-MDD and emotional symptoms/cognitive performance at baseline; Table S6: Demographic, symptom, and cognitive characteristics at baseline and follow-up; Table S7: Associations between changes in perceived stress and cognitive performance; Table S8: Moderation effects of PCS-MDD on the associations between changes in perceived stress and cognitive performance; Table S9: Associations between baseline PCS-MDD and follow-up cognitive performance; Tables S10–S13: Sensitivity analyses of alternative PCS templates with anxiety and depressive symptoms; Tables S14–S15: Demographics and clinical characteristics in the clinical sample, including MDD and bipolar depression comparisons. Figure S1: MDD-specific CSS map; Figure S2: Connection weights in the MDD-specific CSS map; Figure S3: Distribution of age at baseline and follow-up, and the interval between assessments; Figure S4: Changes in perceived stress, emotional symptoms, and cognitive performance; Figure S5: Exploratory analyses results.

## Data Availability

Neuroimaging data from the QTAB cohort are publicly available via OpenNeuro (QTAB dataset) [53]. Non-imaging phenotypic data are available through the Zenodo data-sharing platform under restricted access [54]. Neuroimaging and clinical data from the independent clinical cohort are not publicly available due to privacy and ethical restrictions but can be accessed upon reasonable request to the corresponding author.

## Abbreviations

MDD: Major depressive disorder
CPM: Connectome-based predictive modeling
FSA: Functional segregation analysis
PCS: Polyconnectomic scoring
PCS-MDD: Polyconnectomic scoring for major depressive disorder
QTAB: Queensland Twin Adolescent Brain project
MRI: Magnetic resonance imaging
fMRI: Functional magnetic resonance imaging
HREC: Human Research Ethics Committee
DPARSF: Data processing assistant for resting-state fMRI
MNI: Montreal Neurological Institute
BOLD: Blood oxygen level-dependent
CSS: Connectome summary statistics
DLSS: Daily Life Stressors Scale
SMFQ: Short Moods and Feelings Questionnaire
SCAS: Spence Children’s Anxiety Scale
NIHTB-CB: National Institutes of Health Toolbox Cognition Battery
LMMs: Linear mixed‐effects models
FD: Framewise displacement
SD: Standard deviation
PCS-Anxiety: Polyconnectomic scoring for anxiety disorder
PCS-Bipolar: Polyconnectomic scoring for bipolar disorder
PCS-SCZ: Polyconnectomic scoring for schizophrenia
M.I.N.I. Kid: Mini International Neuropsychiatric Interview for Children and Adolescents
MADRS: Montgomery–Åsberg Depression Rating Scale
PSS-14: Perceived Stress Scale-14
ANCOVA: Analysis of covariance
