# Supplementary material for: Neural vulnerability to stress in adolescents: a longitudinal study using polyconnectomic scoring of depression risk
Source: BMC Med. 2026 Feb 24;24:184. doi: 10.1186/s12916-026-04704-3 (PMC13037335; doi:10.1186/s12916-026-04704-3)
Supplement: Supplementary file 1 — Additional file 1: Supplementary methods, results, tables, and figures. Supplementary methods summarize participant characteristics, assessments, and MRI procedures. Supplementary results present analyses of CSS contribution patterns, PCS-MDD distributions, and cognitive associations. Table S1: Demographic characteristics of participants across four MDD cohorts; Table S2: Top 10 positive and negative connection weights in the MDD-specific CSS map; Table S3: MRI acquisition key parameters for the QTAB and clinical samples; Table S4: Associations between perceived stress and cognitive performance at baseline; Table S5: Associations between PCS-MDD and emotional symptoms/cognitive performance at baseline; Table S6: Demographic, symptom, and cognitive characteristics at baseline and follow-up; Table S7: Associations between changes in perceived stress and cognitive performance; Table S8: Moderation effects of PCS-MDD on the associations between changes in perceived stress and cognitive performance; Table S9: Associations between baseline PCS-MDD and follow-up cognitive performance; Tables S10–S13: Sensitivity analyses of alternative PCS templates with anxiety and depressive symptoms; Tables S14–S15: Demographics and clinical characteristics in the clinical sample, including MDD and bipolar depression comparisons. Figure S1: MDD-specific CSS map; Figure S2: Connection weights in the MDD-specific CSS map; Figure S3: Distribution of age at baseline and follow-up, and the interval between assessments; Figure S4: Changes in perceived stress, emotional symptoms, and cognitive performance; Figure S5: Exploratory analyses results. [file 12916_2026_4704_MOESM1_ESM.docx]

**Neural Vulnerability to Stress in Adolescents: A Longitudinal Study Using Polyconnectomic Scoring of Depression Risk**

**Liu et al.**

**Table of contents**

[1. Supplementary Methods 2](#_Toc221377236)

[1.1 Participants Information 2](#_Toc221377237)

[1.2 MRI Parameters and Preprocessing 3](#_Toc221377238)

[1.3 NIH Toolbox Cognition Battery Tasks 4](#_Toc221377239)

[1.4 Clinical Sample 5](#_Toc221377240)

[1.5 MRI Parameters and Preprocessing of Clinical Sample 5](#_Toc221377241)

[2. Supplementary Results 6](#_Toc221377242)

[2.1 Contribution Patterns in the MDD-Specific CSS Template 6](#_Toc221377243)

[2.2 PCS-MDD in QTAB Compared to Prior Samples 7](#_Toc221377244)

[2.3 Baseline Analyses for Cognition 7](#_Toc221377245)

[2.4 Follow-Up Analyses for Cognition 7](#_Toc221377246)

[3. Supplementary Tables 9](#_Toc221377247)

[Table S1. Demographic Characteristics of Participants Across Four MDD Cohorts 9](#_Toc221377248)

[Table S2. Top 10 Positive and Negative Connection Weights in the MDD-Specific CSS Map 10](#_Toc221377249)

[Table S3. MRI Acquisition Key Parameters for the QTAB and Clinical Samples 11](#_Toc221377250)

[Table S4. Associations Between Perceived Stress and Cognitive Performance at Baseline 12](#_Toc221377251)

[Table S5. Associations Between PCS-MDD and Emotional Symptoms/Cognitive Performance at Baseline 13](#_Toc221377252)

[Table S6. Demographic, Symptom, and Cognitive Characteristics at Baseline and Follow-Up 14](#_Toc221377253)

[Table S7. Associations Between Changes in Perceived Stress and Cognitive Performance 15](#_Toc221377254)

[Table S8. Moderation Effects of PCS-MDD on the Associations Between Changes in Perceived Stress and Cognitive Performance 16](#_Toc221377255)

[Table S9. Associations Between Baseline PCS-MDD and Cognitive Performance at Follow-Up 17](#_Toc221377256)

[Table S10. Associations Between Alternative PCS Templates and Anxiety/Depressive Symptoms at Baseline 18](#_Toc221377257)

[Table S11. Moderation Effects of Alternative PCS Templates on the Associations Between Perceived Stress and Anxiety/Depressive Symptoms at Baseline 19](#_Toc221377258)

[Table S12. Moderation Effects of Alternative PCS Templates on the Associations Between Changes in Perceived Stress and Anxiety/Depressive Symptoms. 20](#_Toc221377259)

[Table S13. Associations Between Alternative Baseline PCS Templates and Anxiety/Depressive Symptoms at Follow-Up 21](#_Toc221377260)

[Table S14. Demographic and Clinical Characteristics in Clinical Sample 22](#_Toc221377261)

[Table S15. Group Comparisons Between MDD and Bipolar Depression in Clinical Sample 23](#_Toc221377262)

[4. Supplementary Figures 24](#_Toc221377264)

[Figure S1. MDD-Specific CSS Map 24](#_Toc221377265)

[Figure S2. Connection Weights in the MDD-Specific CSS Map 25](#_Toc221377266)

[Figure S3. Distribution of Age at Baseline and Follow-Up, and the Interval Between Assessments 26](#_Toc221377267)

[Figure S4. Changes in Perceived Stress, Emotional Symptoms, and Cognitive Performance 27](#_Toc221377268)

[Figure S5. Exploratory Analyses Results 28](#_Toc221377269)

# 1. Supplementary Methods

## 1.1 Participants Information

The Queensland Twin Adolescent Brain Project recruited 422 participants, focusing on twins aged 9 to 13 years who lived within a two-hour travel radius of the Brisbane study center. Participants were identified through the Queensland Twin (QTwin) Register, Twin Research Australia (TRA), and the project’s study website.

Inclusion required both twins in a pair to meet eligibility criteria. Exclusion criteria included serious medical, neurological, or psychiatric conditions (e.g., autism spectrum disorder, attention-deficit/hyperactivity disorder), cardiovascular issues, a history of serious head injury, or cognitive, physical, or sensory impairments that could interfere with study procedures. Additionally, participants were excluded if they had contraindications for magnetic resonance imaging (MRI), such as metal implants or orthodontic braces.

Of the initial cohort, five participants were unable to complete session 1 imaging due to reluctance to enter the MRI system, resulting in an imaging dataset of 417 participants (48% female, mean age 11.3 ± 1.4 years, age range 9.0–14.4 years, 82% right-handed). An additional 10 participants were excluded due to missing functional magnetic resonance imaging (fMRI) data, leaving a final sample of 407 participants for analysis.

## 1.2 MRI Parameters and Preprocessing

Structural and functional imaging data were collected using a 3T Magnetom Prisma scanner (Siemens Medical Solutions, Erlangen) equipped with a 64-channel head coil at the Centre for Advanced Imaging, University of Queensland. Resting-state fMRI (rs-fMRI): Imaging parameters included a repetition time (TR) of 930 ms, echo time (TE) of 30 ms, flip angle (FA) of 52°, 72 slices, field of view (FOV) of 208 × 208 mm, slice acceleration factor of 6, and 327 volumes per scan. Each scan lasted 5 minutes and 13 seconds. High-Resolution Structural Imaging: Structural images were acquired with a TR of 4000 ms, TE of 2.99 ms, inversion times (TI1/TI2) of 700 ms and 2220 ms, flip angles (FA1/FA2) of 6° and 7°, 192 slices, FOV of 256 × 240 mm, and in-plane acceleration factor of 3. Each scan had a duration of 6 minutes and 4 seconds.

MRI preprocessing was conducted using the DPARSF toolbox (http://www.rfmri.org/) and included the following steps: removal of the first 10 volumes from each functional scan, correction for head motion, and covariate regression to account for white matter signals, cerebrospinal fluid signals, and Friston-24 motion parameters. Global signal regression was omitted due to ongoing debates about its potential effects. The images were then normalized to the Montreal Neurological Institute (MNI) space with a resolution of 3 mm × 3 mm × 3 mm and underwent temporal bandpass filtering (0.01–0.10 Hz).

## 1.3 NIH Toolbox Cognition Battery Tasks

**1) Flanker Inhibitory Control and Attention Test (Flanker)**

The Flanker test assesses inhibitory control and attention by presenting a central target arrow flanked by congruent or incongruent arrows. Participants are required to indicate the direction of the central arrow. Both accuracy and reaction time are recorded, and a composite score integrating these metrics is generated using the NIH Toolbox scoring algorithm to yield fully corrected T-scores.

**2) Dimensional Change Card Sort Test (DCCS)**

The DCCS evaluates cognitive flexibility and executive functioning by requiring participants to match stimuli to target cards based on either shape or color. Task rules shift according to task instructions across trials, necessitating adaptive responses. Performance is indexed by a score derived from accuracy and reaction time and converted into fully corrected T-scores.

**3)** **Pattern Comparison Processing Speed Test**

This task measures processing speed by presenting pairs of visual patterns and asking participants to judge whether the patterns are identical or different as quickly as possible within a 90-second time limit. Performance is quantified by the number of correct responses and transformed into fully corrected T-scores.

**4) Picture Sequence Memory Test (PSMT)**

The PSMT assesses episodic memory by presenting participants with sequences of pictures depicting meaningful activities, which must be recalled and reordered from memory. Sequence length increases across trials, and scoring is based on sequence accuracy, yielding fully corrected T-scores.

**5)** **Picture Vocabulary Test (PVT)**

The PVT evaluates receptive vocabulary and language ability by presenting four images and requiring participants to select the image that best corresponds to a spoken word. Item difficulty is modeled using item response theory (IRT), and responses are converted into fully corrected T-scores.

**6) Oral Reading Recognition Test (ORRT)**

The ORRT measures reading decoding and language skills by having participants read letters and words aloud from a screen. Task difficulty adapts dynamically based on performance, and scores are derived from correct responses using an IRT-calibrated model to produce fully corrected T-scores.

## 1.4 Clinical Sample

This study explored perceived stress and emotional distress in adolescents diagnosed with major depressive disorder (MDD) or experiencing a depressive episode of Bipolar II Disorder, utilizing the clinical sample of 80 adolescents recruited from Tianjin Anding Hospital. Participants were aged 12 to 18 years and included if they met the diagnostic criteria outlined in the Mini International Neuropsychiatric Interview for Children and Adolescents (M.I.N.I. Kid) 5.0 Revision and had a Montgomery–Åsberg Depression Rating Scale (MADRS) score of 22 or higher. Exclusion criteria included the presence of psychotic symptoms, metal implants located above the chest (particularly in the intracranial or cardiac regions), neurological disorders, brain diseases, severe physical conditions, a history of traumatic brain injury or surgery, repetitive transcranial magnetic stimulation (rTMS) or electroconvulsive therapy (ECT) within the last 6 months, drug or alcohol abuse within the past year, inability to communicate or cooperate, concurrent participation in other clinical trials, and a Young Mania Rating Scale (YMRS) score greater than 8.

## 1.5 MRI Parameters and Preprocessing of Clinical Sample

Structural and functional imaging data were acquired using a 3T Magnetom Prisma scanner (Siemens Medical Solutions, Erlangen, Germany) equipped with a 64-channel head coil. Participants were instructed to remain awake with their eyes closed and avoid head motion during the scanning process. rs-fMRI: Imaging parameters included a TR of 800 ms, TE of 30 ms, FA of 56°, 72 slices with a thickness of 2 mm, a matrix size of 104 × 104, and a voxel size of 2 × 2 × 2 mm. The scanning protocol captured 450 time points. High-Resolution Structural Imaging: Structural images were acquired with a TR of 2000 ms, TE of 2.32 ms, FA of 8°, 208 slices with a thickness of 0.9 mm, a matrix size of 256 × 256, and a voxel size of 0.9 × 0.9 × 0.9 mm.

MRI preprocessing followed the same procedures as those implemented in the QTAB cohort, ensuring consistency in data processing across studies.

# 2. Supplementary Results

## 2.1 Contribution Patterns in the MDD-Specific CSS Template

We extracted the CSS weights for all region-to-region connections and identified the most strongly weighted positive and negative contributors to PCS-MDD. The connections with the highest positive weights predominantly involved interactions between the sensorimotor networks (SMN) and salience/ventral attention networks, or between the SMN and frontoparietal control network. Notable examples include: 1) LH_SomMot_10 – RH_SalVentAttn_TempOccPar_7; 2) LH_SomMot_18 – LH_Cont_Par_2; 3) LH_SomMot_36 – RH_SalVentAttn_TempOccPar_7.

In contrast, the connections with the highest negative weights were primarily within the SMN or between the SMN and visual networks, such as: 1) LH_SomMot_13 – RH_SomMot_40; 2) LH_SomMot_14 – RH_SomMot_40; 3) LH_Vis_20 – LH_SomMot_6. A comprehensive list of edge weights and corresponding brain regions and networks is provided in Table S2 and Figure S2. These patterns remained consistent across multiple thresholds (top 10/20 edges), indicating stable dominant contributors to the CSS architecture.

## 2.2 PCS-MDD in QTAB Compared to Prior Samples

We compared the distributions of PCS-MDD in the current QTAB cohort with those from prior adolescent samples (MDDII) (Libedinsky I, et al., Biol Psychiatry, 2025). PCS-MDD values in the QTAB cohort (-0.0011 ± 0.00) were comparable to MDDII control participants (-0.0016 ± 0.00) and markedly lower than those observed in MDDII patients (0.0013± 0.01).

## 2.3 Baseline Analyses for Cognition

1) Negative associations were identified between DLSS and Pattern Comparison (*β* = -0.12, *p* = 0.010), Picture Sequence (*β* = -0.11, *p* = 0.033), and Oral Reading Recognition (*β* = -0.11, *p* = 0.021). After applying Bonferroni correction, no significant associations between DLSS and cognitive measures.

2) The PCS-MDD score was negatively associated with Picture Vocabulary (*β* = -0.10, *p* = 0.038). After applying Bonferroni correction, no significant associations between PCS-MDD and cognitive measures.

## 2.4 Follow-Up Analyses for Cognition

1) Change Score in Cognition: Significant improvements were observed in Flanker, Card Sort, Picture Sequence, Oral Reading Recognition, and Pattern Comparison scores, whereas Picture Vocabulary scores declined.

2) Association Analyses: Negative associations were identified between baseline PCS-MDD and Picture Vocabulary (*β* = -0.12, *p* = 0.032) at follow-up (Table S10). After applying Bonferroni correction, no significant associations between PCS-MDD and cognitive measures.

# 3. Supplementary Tables

## **Table S1.** Demographic Characteristics of Participants Across Four MDD Cohorts

| Cohort | Group | N | Sex (M/F) | Age (mean ± SD, years) |
| --- | --- | --- | --- | --- |
| MDDI | Control | 878 | 386/492 | 36.66 ± 15.6 |
| MDDI | Patient | 240 | 114/126 | 42.06 ± 12.0 |
| MDDII | Control | 46 | 23/23 | 15.36 ± 0.9 |
| MDDII | Patient | 55 | 44/11 | 15.56 ± 0.8 |
| MDDIII | Control | 178 | 81/97 | 10.76 ± 3.4 |
| MDDIII | Patient | 89 | 50/39 | 14.46 ± 3.3 |
| MDDIV | Control | 504 | 329/175 | 37.26 ± 13.4 |
| MDDIV | Patient | 500 | 341/159 | 37.26 ± 13.1 |

***Abbreviations:*** MDD, major depressive disorders; M, male; F, female.

***Note:*** Detailed information was available from the previous study (Libedinsky I, et al., Polyconnectomic Scoring of Functional Connectivity Patterns Across Eight Neuropsychiatric and Three Neurodegenerative Disorders, Biol Psychiatry, 2025).

## **Table S2.** Top 10 Positive and Negative Connection Weights in the MDD-Specific CSS Map

| Brian Regions | Brian Regions | Weight |
| --- | --- | --- |
| LH_SomMot_10 | RH_SalVentAttn_TempOccPar_7 | 0.268 |
| LH_Default_Temp_8 | LH_Default_pCunPCC_5 | 0.266 |
| LH_SomMot_18 | LH_Cont_Par_2 | 0.265 |
| LH_SomMot_36 | RH_SalVentAttn_TempOccPar_7 | 0.265 |
| RH_SomMot_3 | RH_Cont_PFCl_4 | 0.264 |
| LH_SomMot_33 | LH_Cont_Par_2 | 0.256 |
| RH_SomMot_32 | RH_SalVentAttn_TempOccPar_7 | 0.253 |
| RH_SomMot_7 | RH_Cont_PFCl_4 | 0.247 |
| LH_SalVentAttn_PFCl_1 | RH_SomMot_38 | 0.246 |
| LH_SomMot_18 | RH_SalVentAttn_TempOccPar_7 | 0.243 |
| RH_SomMot_10 | RH_SalVentAttn_TempOccPar_7 | 0.243 |
| LH_Vis_20 | LH_SomMot_6 | -0.263 |
| RH_SomMot_9 | RH_SomMot_14 | -0.263 |
| LH_SomMot_13 | RH_SomMot_32 | -0.266 |
| RH_SomMot_5 | RH_SomMot_24 | -0.275 |
| LH_SomMot_14 | LH_SomMot_36 | -0.276 |
| RH_SomMot_16 | RH_SomMot_40 | -0.285 |
| LH_SomMot_13 | LH_SomMot_32 | -0.287 |
| LH_SomMot_12 | RH_SomMot_40 | -0.287 |
| LH_SomMot_13 | RH_SomMot_40 | -0.321 |
| LH_SomMot_14 | RH_SomMot_40 | -0.326 |

***Abbreviations:*** CSS, connectome summary statistics; MDD, major depressive disorder;

LH, left hemisphere; RH, right hemisphere; Cont, Frontoparietal Control Network; Default, Default Mode Network; SomMot, Somatomotor Network; SalVentAttn, Salience/Ventral Attention Network; Vis, Visual Network; DorsAttn, Dorsal Attention Network; Limbic, Limbic Network.

## **Table S3.** MRI Acquisition Key Parameters for the QTAB and Clinical Samples

| Parameter | QTAB Sample | Clinical Sample |
| --- | --- | --- |
| Scanner | 3T Magnetom Prisma (Siemens) | 3T Magnetom Prisma (Siemens) |
| Head coil | 64-channel | 64-channel |
| Resting-state fMRI |  |  |
| Repetition time | 930 ms | 800 ms |
| Echo time | 30 ms | 30 ms |
| Flip angle | 52° | 56° |
| Slices | 72 slices | 72 slices |
| Volumes / Time points | 327 volumes | 450 volumes |
| High-resolution structural imaging | |  |
| Repetition time | 4000 ms | 2000 ms |
| Echo time | 2.99 ms | 2.32 ms |
| Flip angle | 6° / 7° | 8° |
| Slices | 192 slices | 208 slices |

***Abbreviations:*** QTAB: Queensland Twin Adolescent Brain.

## Table S4. Associations Between Perceived Stress and Cognitive Performance at Baseline

| Outcome | β | 95% CI | p |
| --- | --- | --- | --- |
| Flanker | –0.01 | –0.11, 0.09 | 0.8 |
| Card Sort | –0.07 | –0.16, 0.03 | 0.2 |
| Pattern Comparison | –0.12 | –0.22, –0.03 | 0.01 |
| Picture Sequence | –0.10 | –0.20, –0.01 | 0.033 |
| Picture Vocabulary | –0.06 | –0.15, 0.04 | 0.25 |
| Oral Reading Recognition | –0.11 | –0.20, –0.02 | 0.021 |

***Abbreviations:*** 95% CI, 95% confidence interval.

## Table S5. Associations Between PCS-MDD and Emotional Symptoms/Cognitive Performance at Baseline

| Domain | Outcome | β | 95% CI | p |
| --- | --- | --- | --- | --- |
| Symptoms | SCAS | 0.03 | –0.06, 0.12 | 0.5 |
|  | SMFQ | 0.003 | –0.09, 0.10 | 0.94 |
| Cognition | Flanker | –0.01 | –0.11, 0.08 | 0.81 |
|  | Card Sort | –0.07 | –0.17, 0.03 | 0.17 |
|  | Pattern Comparison | –0.01 | –0.10, 0.09 | 0.9 |
|  | Picture Sequence | –0.09 | –0.19, 0.001 | 0.054 |
|  | Picture Vocabulary | –0.10 | –0.19, –0.01 | 0.038 |
|  | Oral Reading Recognition | –0.04 | –0.13, 0.05 | 0.41 |

***Abbreviations:*** PCS-MDD, polyconnectomic scoring for major depressive disorders; SCAS, Spence Children’s Anxiety Scale; SMFQ, Short Moods and Feelings Questionnaire; 95% CI, 95% confidence interval.

| Characteristics | Baseline (n = 407) | Follow-up (n = 298) |
| --- | --- | --- |
| Sex (female/male) | 197/210 | 153/145 |
| Age (years) | 11.32 ± 1.34 | 13.03 ± 1.50 |
| Handedness (Left/Right) | 72/335 | 46/252 |
| Stress |  |  |
| DLSS score | 22.10 ± 11.63 | 24.53 ± 13.20 |
| Anxiety and depression |  |  |
| SCAS score | 24.79 ± 13.94 | 24.74 ± 14.82 |
| SMFQ score | 4.25 ± 3.47 | 4.85 ± 4.14 |
| Cognition |  |  |
| Flanker | 98.27 ± 15.47 | 106.05 ± 18.29 |
| Card Sort | 100.87 ± 19.84 | 113.21 ± 21.57 |
| Pattern Comparison | 98.55 ± 21.25 | 116.20 ± 20.48 |
| Picture Sequence | 101.37 ± 16.16 | 108.40 ± 16.93 |
| Picture Vocabulary | 103.72 ± 13.56 | 101.51 ± 12.82 |
| Oral Reading Recognition | 106.18 ± 15.97 | 107.29 ± 15.14 |

## Table S6. Demographic, Symptom, and Cognitive Characteristics at Baseline and Follow-Up

***Abbreviations:*** DLSS, Daily Life Stressors Scale; SCAS, Spence Children’s Anxiety Scale; SMFQ, Short Moods and Feelings Questionnaire.

## Table S7. Associations Between Changes in Perceived Stress and Cognitive Performance

| Cognitive Outcome | β | 95% CI | p |
| --- | --- | --- | --- |
| Flanker change | 0.02 | -0.10, 0.13 | 0.758 |
| Card Sort change | -0.05 | -0.16, 0.07 | 0.414 |
| Pattern Comparison change | -0.10 | -0.22, 0.02 | 0.094 |
| Picture Sequence change | -0.06 | -0.17, 0.06 | 0.344 |
| Picture Vocabulary change | 0.03 | -0.08, 0.15 | 0.554 |
| Oral Reading Recognition change | 0.00 | -0.11, 0.12 | 0.939 |

***Abbreviations:*** 95% CI, 95% confidence interval.

## Table S8. Moderation Effects of PCS-MDD on the Associations Between Changes in Perceived Stress and Cognitive Performance

| Outcome | β | 95% CI | p | ΔR² |
| --- | --- | --- | --- | --- |
| Flanker change | -0.07 | -0.19, 0.05 | 0.274 | 0.004 |
| Card Sort change | -0.12 | -0.24, 0.00 | 0.052 | 0.012 |
| Pattern Comparison change | 0.05 | -0.07, 0.17 | 0.407 | 0.002 |
| Picture Sequence change | -0.04 | -0.16, 0.08 | 0.526 | 0.001 |
| Picture Vocabulary change | -0.12 | -0.24, 0.00 | 0.055 | 0.012 |
| Oral Reading Recognition change | -0.02 | -0.14, 0.10 | 0.776 | 0.000 |

***Abbreviations:*** PCS-MDD, polyconnectomic scoring for major depressive disorders; 95% CI, 95% confidence interval.

## Table S9. Associations Between Baseline PCS-MDD and Follow-Up Cognitive Performance

| Outcome | β | 95% CI | p |
| --- | --- | --- | --- |
| Flanker change | -0.04 | -0.16, 0.07 | 0.48 |
| Card Sort change | -0.05 | -0.16, 0.07 | 0.424 |
| Pattern Comparison change | 0.04 | -0.07, 0.15 | 0.498 |
| Picture Sequence change | 0 | -0.11, 0.11 | 0.993 |
| Picture Vocabulary change | -0.11 | -0.22, -0.01 | 0.032 |
| Oral Reading Recognition change | -0.05 | -0.14, 0.05 | 0.358 |

***Abbreviations:*** PCS-MDD, polyconnectomic scoring for major depressive disorders; 95% CI, 95% confidence interval.

## Table S10. Associations Between Alternative PCS Templates and Anxiety/Depressive Symptoms at Baseline

| Predictor | Outcome | β | 95% CI | p |
| --- | --- | --- | --- | --- |
| PCS-Anxiety | SCAS | 0.07 | -0.02, 0.17 | 0.121 |
| PCS-Anxiety | SMFQ | 0.03 | -0.06, 0.13 | 0.478 |
| PCS-Bipolar | SCAS | 0.00 | -0.10, 0.09 | 0.976 |
| PCS-Bipolar | SMFQ | -0.03 | -0.13, 0.06 | 0.512 |
| PCS-SCZ | SCAS | -0.05 | -0.14, 0.04 | 0.282 |
| PCS-SCZ | SMFQ | -0.04 | -0.14, 0.05 | 0.35 |

***Abbreviations:*** PCS-Anxiety, polyconnectomic scoring for anxiety disorder; PCS-Bipolar, polyconnectomic scoring for bipolar disorder; PCS-SCZ, polyconnectomic scoring for schizophrenia; SCAS, Spence Children’s Anxiety Scale; SMFQ, Short Moods and Feelings Questionnaire; 95% CI, 95% confidence interval.

## Table S11. Moderation Effects of Alternative PCS Templates on the Associations Between Perceived Stress and Anxiety/Depressive Symptoms at Baseline

| Moderator | Outcome | β | 95% CI | p | ΔR² |
| --- | --- | --- | --- | --- | --- |
| PCS-Anxiety | SCAS | 0.00 | -0.08, 0.08 | 0.984 | -0.001 |
| PCS-Anxiety | SMFQ | 0.05 | -0.03, 0.14 | 0.215 | 0.001 |
| PCS-Bipolar | SCAS | -0.01 | -0.08, 0.06 | 0.757 | -0.001 |
| PCS-Bipolar | SMFQ | 0.01 | -0.06, 0.09 | 0.696 | -0.001 |
| PCS-SCZ | SCAS | 0.01 | -0.07, 0.08 | 0.853 | 0.000 |
| PCS-SCZ | SMFQ | 0.07 | -0.01, 0.15 | 0.092 | 0.004 |

***Abbreviations:*** PCS-Anxiety, polyconnectomic scoring for anxiety disorder; PCS-Bipolar, polyconnectomic scoring for bipolar disorder; PCS-SCZ, polyconnectomic scoring for schizophrenia; SCAS, Spence Children’s Anxiety Scale; SMFQ, Short Moods and Feelings Questionnaire; 95% CI, 95% confidence interval.

| Moderator | Outcome | β | 95% CI | p | ΔR² |
| --- | --- | --- | --- | --- | --- |
| PCS-Anxiety | SCAS change | 0.08 | -0.03, 0.18 | 0.156 | 0.002 |
| PCS-Anxiety | SMFQ change | 0.08 | -0.03, 0.19 | 0.132 | 0.003 |
| PCS-Bipolar | SCAS change | 0.15 | 0.04, 0.25 | 0.006 | 0.018 |
| PCS-Bipolar | SMFQ change | 0.09 | -0.02, 0.20 | 0.113 | 0.005 |
| PCS-SCZ | SCAS change | 0.04 | -0.06, 0.13 | 0.424 | 0.001 |
| PCS-SCZ | SMFQ change | 0.04 | -0.06, 0.13 | 0.442 | 0.001 |

## Table S12. Moderation Effects of Alternative PCS Templates on the Associations Between Changes in Perceived Stress and Anxiety/Depressive Symptoms.

***Abbreviations:*** PCS-Anxiety, polyconnectomic scoring for anxiety disorder; PCS-Bipolar, polyconnectomic scoring for bipolar disorder; PCS-SCZ, polyconnectomic scoring for schizophrenia; SCAS, Spence Children’s Anxiety Scale; SMFQ, Short Moods and Feelings Questionnaire; 95% CI, 95% confidence interval.

## Table S13. Associations Between Alternative Baseline PCS Templates and Follow-Up Anxiety/Depressive Symptoms

| Predictor | Outcome | β | 95% CI | p |
| --- | --- | --- | --- | --- |
| PCS-Anxiety ^a^ | SCAS ^b^ | -0.01 | -0.11, 0.10 | 0.864 |
| PCS-Anxiety ^a^ | SMFQ ^b^ | 0.04 | -0.07, 0.15 | 0.439 |
| PCS-Bipolar ^a^ | SCAS ^b^ | 0.04 | -0.07, 0.15 | 0.516 |
| PCS-Bipolar ^a^ | SMFQ ^b^ | -0.03 | -0.15, 0.08 | 0.595 |
| PCS-SCZ ^a^ | SCAS ^b^ | 0.06 | -0.04, 0.17 | 0.229 |
| PCS-SCZ ^a^ | SMFQ ^b^ | -0.01 | -0.12, 0.10 | 0.826 |

***Abbreviations:*** PCS-Anxiety, polyconnectomic scoring for anxiety disorder; PCS-Bipolar, polyconnectomic scoring for bipolar disorder; PCS-SCZ, polyconnectomic scoring for schizophrenia; SCAS, Spence Children’s Anxiety Scale; SMFQ, Short Moods and Feelings Questionnaire; 95% CI, 95% confidence interval.

***Note:*** ^a^ means baseline; ^b^ means follow-up.

## **Table S14**. Demographic and Clinical Characteristics in Clinical Sample

|  | Total  (n = 80) | Low PCS-MDD  (n = 26) | Medium PCS-MDD  (n = 26) | High PCS-MDD  (n = 28) |
| --- | --- | --- | --- | --- |
| Sex (female/male) | 61/19 | 19/7 | 20/6 | 22/6 |
| Age (years) | 14.91 ± 2.19 | 14.88 ± 1.70 | 15.35 ± 1.20 | 14.54 ± 3.12 |
| Participants types (MDD/bipolar depression) | 63/17 | 21/5 | 20/6 | 22/6 |
| PSS-14 score | 54.48 ± 7.53 | 54.88 ± 7.48 | 51.92 ± 7.24 | 56.46 ± 7.40 |
| MARDS score | 29.03 ± 6.20 | 27.15 ± 5.27 | 28.92 ± 5.25 | 30.86 ± 7.37 |

***Abbreviations:*** PSS-14, Perceived Stress Scale-14; MADRS, Montgomery–Åsberg Depression Rating Scale; PCS-MDD, polyconnectomic scoring for major depressive disorder.

|  | MDD  (n = 63) | Bipolar depression (n = 17) | χ^2^/t | p |
| --- | --- | --- | --- | --- |
| Sex (female/male) | 49/14 | 12/5 | 0.382 | 0.536 |
| Age (years) | 14.52 ± 2.23 | 16.35 ± 1.32 | -3.227 | 0.002 |
| PSS-14 score | 54.49 ± 7.89 | 54.41 ± 6.18 | 0.039 | 0.969 |
| MARDS score | 28.95 ± 6.25 | 29.29 ± 6.20 | -0.200 | 0.842 |
| PCS-MDD | 2.01 ± 0.83 | 2.06 ± 0.83 | -0.189 | 0.851 |

## Table S15**. Group Comparisons Between MDD and Bipolar Depression in Clinical Sample**

*Abbreviations:* PSS-14, Perceived Stress Scale-14; MADRS, Montgomery–Åsberg Depression Rating Scale; PCS-MDD, polyconnectomic scoring for major depressive disorder.

# **4. Supplementary Figures**


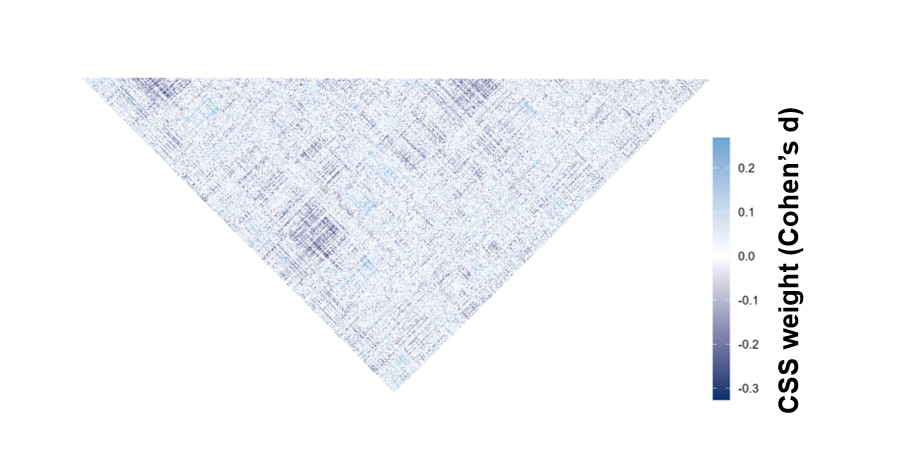


## Figure S1. MDD-Specific CSS Map

***Abbreviations:*** CSS, connectome summary statistics; MDD, major depressive disorder.


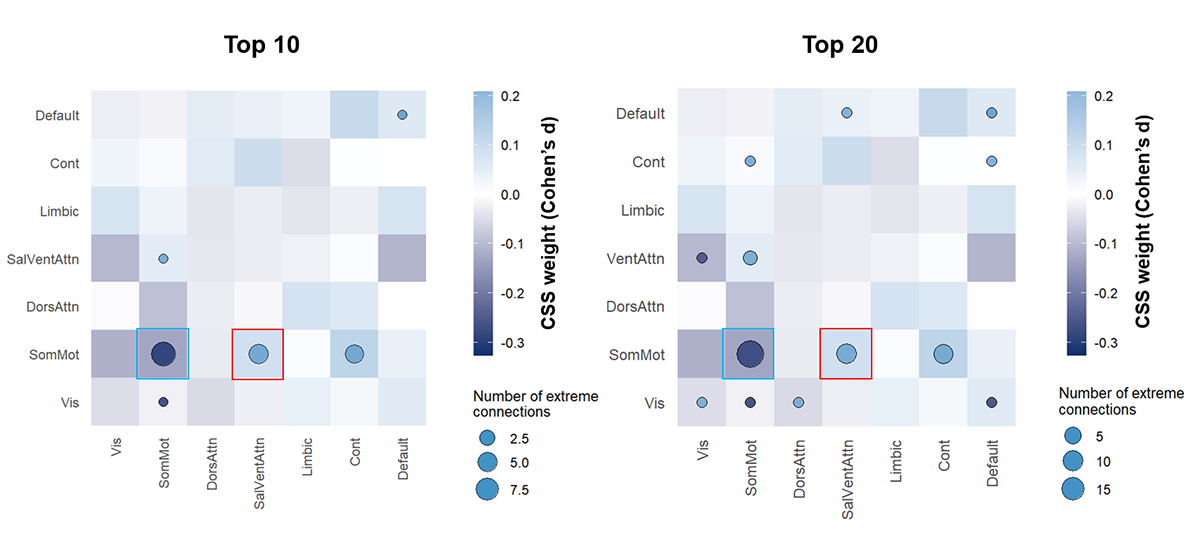


## Figure S2. Connection Weights in the MDD-Specific CSS Map

***Abbreviations:*** Cont, Frontoparietal Control Network; Default, Default Mode Network; SomMot, Somatomotor Network; SalVentAttn, Salience/Ventral Attention Network; Vis, Visual Network; DorsAttn, Dorsal Attention Network; Limbic, Limbic Network.

***Note:*** Color denotes the CSS weight (Cohen’s d) for each connection, with deep blue indicating negative values and light blue indicating positive values. Point size represents the number of extreme connections within the top 10/20 (positive or negative).


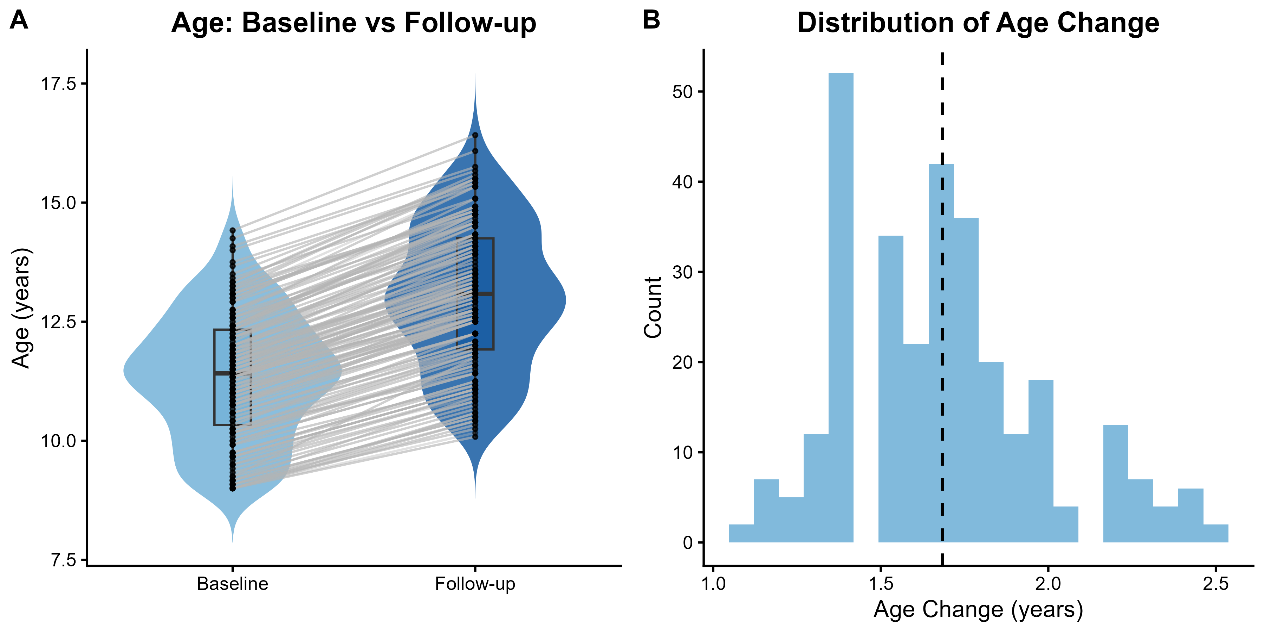


## Figure S3. Distribution of Age at Baseline and Follow-Up, and the Interval Between Assessments


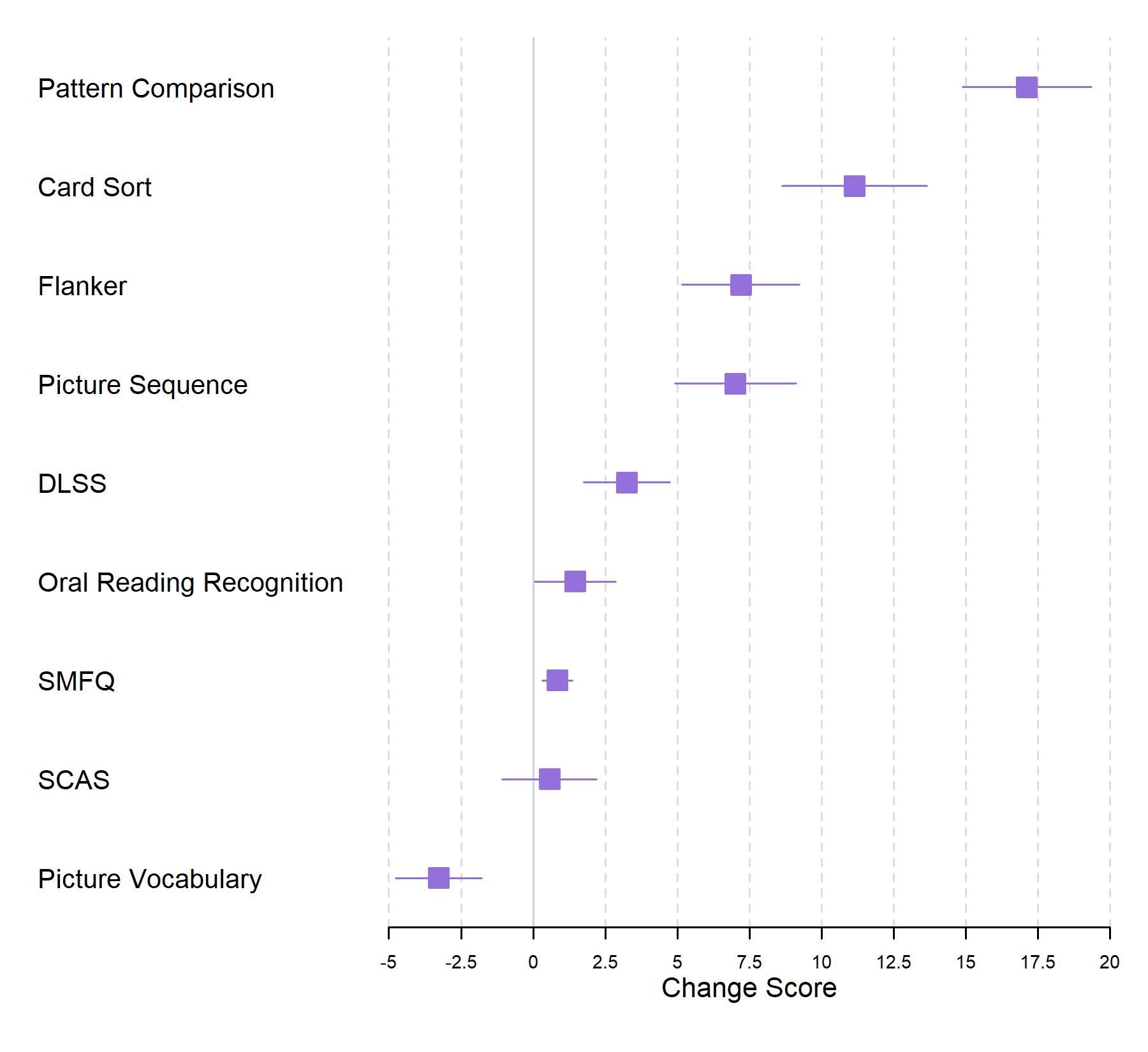


## Figure S4. Changes in Perceived Stress, Emotional Symptoms, and Cognitive Performance

***Abbreviations:*** SCAS, Spence Children’s Anxiety Scale; SMFQ, Short Moods and Feelings Questionnaire; PCS-MDD, polyconnectomic scoring for major depressive disorder.


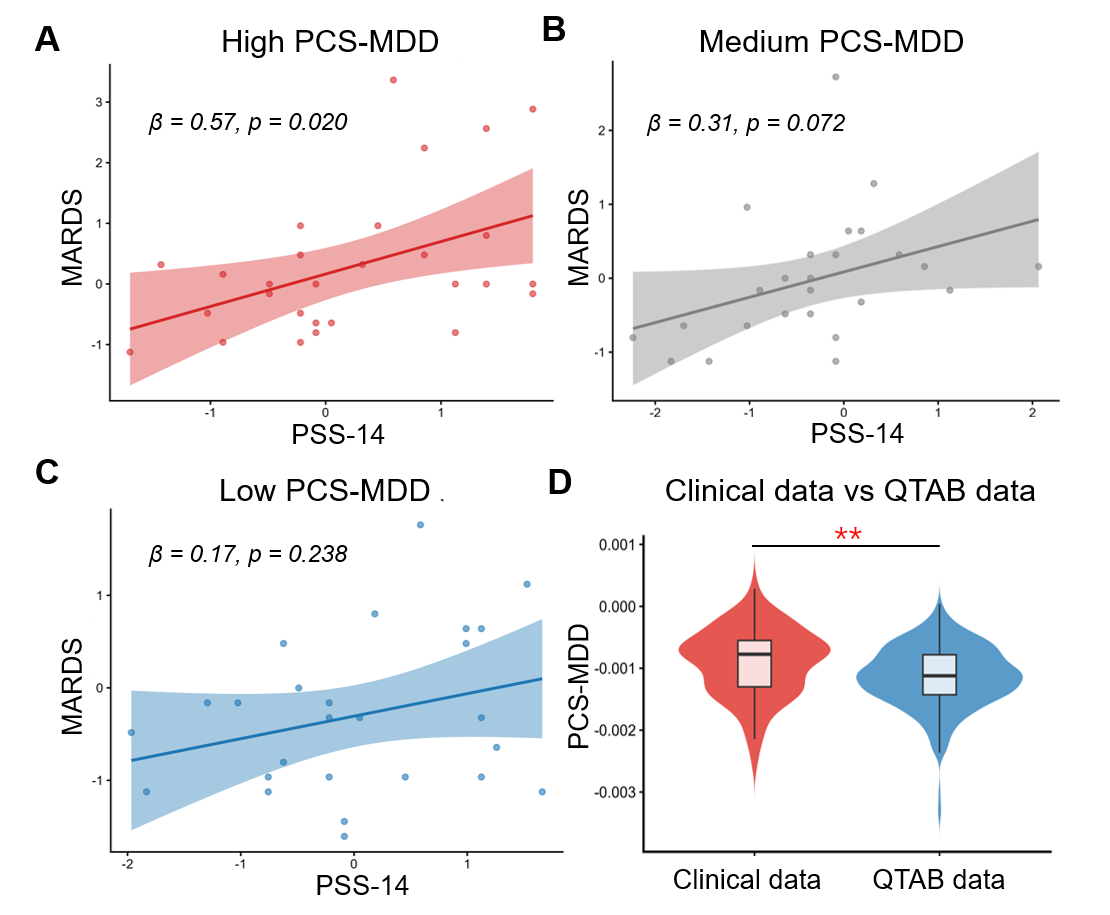


## Figure S5. Exploratory Analyses Results

(A-C) Associations Between Perceived Stress and Depressive Symptoms Across Different PCS-MDD Levels.

(D) PCS-MDD in QTAB Data and Clinical Data.

***Abbreviations:*** PSS-14, Perceived Stress Scale-14; MADRS, Montgomery–Åsberg Depression Rating Scale; PCS-MDD, polyconnectomic scoring for major depressive disorder; QTAB, Queensland Twin Adolescent Brain project.

***Note:*** ^**^ *p* < 0.01.
